# Past and present genetic structure of the tropical rainforest palm *Astrocaryum mexicanum*: effects of anthropogenic fragmentation

**DOI:** 10.7717/peerj.19784

**Published:** 2026-01-13

**Authors:** Jorge O. Juárez-Ramírez, Juan Núñez-Farfán

**Affiliations:** Lab. of Ecological Genetics and Evolution, Department of Evolutionary Ecology, Institute of Ecology, Universidad Nacional Autónoma de México, Ciudad de México, Distrito Federal, Mexico

**Keywords:** Lowland tropical rain forest, *Astrocaryum mexicanum*, Ancestral population bottleneck, Population genetic structure, Keystone species, Forest fragmentation, Last glacial maximum, New microsatellites for palms, Pre- and post-fragmentation genetic structure

## Abstract

To assess whether fragmentation of the lowland rainforest of *Los Tuxtlas* natural reserve has altered the genetic structure of understory palm *Astrocaryum mexicanum*, we analyzed populations from undisturbed forest and forest fragments. The questions that this study addressed were: Has habitat fragmentation reduced gene flow and within-population genetic variation (allele loss)? Has this process, in turn, increased population genetic differentiation of populations in fragments? We expected that reduced population sizes and gene flow, in fragments, has increased the effects of genetic drift, thus affecting genetic structure. The design of the study allows control for pre-fragmentation genetic structure, a common criticism against fragmentation studies, and addresses this question for a community-level important palm tree species of the tropical rain forests of southern Mexico. We sampled two cohorts (*i.e.*, pre- and post-fragmentation palms) in each of eight populations (thirty individuals per cohort or generation, and a separation of four to nine km between populations), one composed by adult palms (80–140 years old), and the other of seedlings <3 years old. We estimated *R_ST_*, inbreeding coefficient, number of alleles, heterozygosity, linkage disequilibrium, and number of migrants per generation, using variation at eight novel microsatellite loci, developed *ex profeso* for this study. Results indicate lack of differentiation between population pairs, and most genetic variation exists within subpopulations, implying high historical connectivity. Fragments were not genetically distinct from continuous forest populations. Simulations suggest a severe effective population size reduction at the outset of the Last Glacial Maximum 26,000 YBP, after which the area was recolonized by individuals from Central America. It is possible that the number of reproductive events that have passed since the onset of fragmentation has been insufficient to detect an effect on genetic variation, or that the extant number of palm trees in fragments is high enough to maintain the genetic diversity; bottleneck simulations agree with the first explanation. Notwithstanding, evidence suggests that populations in fragments face harsher environmental conditions, selecting against homozygotes, a situation that can jeopardize their persistence in fragments if population size is too small.

## Introduction

Human-induced fragmentation of terrestrial habitats is one of the most serious threats for the persistence of natural populations (Fahrig, 2003). Changing land use to agriculture and cattle ranching, as well as irrational exploitation of forest products, result in the reduction of primeval forest areas and isolation of populations within small-sized fragments that remain surrounded by a matrix of disturbed habitat (Wilcove, McLellan & Dobson, 1986). Forest fragmentation changes local environmental physical variables, like wind flow, light incidence, air humidity and temperature (Pinto et al., 2010), and may change ecological interactions between species (Laurance, 2005). For many plant species the ecological changes induced by fragmentation might even lead to local extinction, if abiotic conditions would change beyond plants’ tolerance limits, and/or if specialized mutualists such as pollinators (Bond, 1994) are lost or have their effectiveness compromised.

An important assumption is that fragmentation restricts gene flow between population in fragments (Chávez-Pesqueira et al., 2015). Changes in population size and in the direction and magnitude of gene flow among populations may alter the amount and distribution of genetic variation among populations inhabiting both primeval and fragmented landscapes. For instance, it is expected that the isolation of small subsets of plants would increase their level of inbreeding (Young, Boyle & Brown, 1996), and inbreeding can increase in progenies relative to adult plants in tropical tree species due to changes in plants’ mating system brought about by forest fragmentation (Lowe et al., 2005). Thus, assessing the impact of forest fragmentation on genetic parameters like heterozygosis of natural populations is important, especially in outcrossing species since an increase in selfing may cause increased risk of extinction. Another expected effect of fragmentation is the reduction of within population (*i.e.,* fragment) genetic variation, and the increase of inter-population variation by genetic drift (Wright, 1984).

Disagreement between theoretical expectations and some empirical results regarding the effects of fragmentation on genetic diversity and structure of plant populations, particularly in long-lived species, has spurred criticism (see Kramer et al., 2008). In long-lived species, the time since fragmentation may not be long enough to detect changes in their genetic structure. Furthermore, even if extant populations are decimated, researchers should control for populations, genetic structure prior to fragmentation. A review of evidence gathered for neotropical trees detected a significant increase in genetic differentiation and inbreeding brought about by fragmentation, regardless the plant species’ generation time (Lowe et al., 2005).

Understory plants, including several palm lineages, are not well represented among the studies of genetic consequences of habitat fragmentation (see Suárez-Montes, Chávez-Pesqueira & Núñez Farfán, 2016), despite their importance in microclimate maintenance and ecological interactions (Arroyo-Rodríguez et al., 2007; Cunningham, 1997; Piñero, Sarukhán & González, 1977; Sloan, Newell & Ellison, 1991). On the other hand, understory palms have reproductive traits that make them potentially susceptible to habitat fragmentation (Lande & Schemske, 1985; Eguiarte et al., 1993). In the last 13 years, three published meta-analyses analyzing the impact (or lack thereof) of fragmentation on genetic diversity of plants, factoring in life history traits present in many understory palms (Vranckx et al., 2011; Schlaepfer et al., 2018; González, Gómez-Silva & Ramírez, 2019), have detected negative effects. In recent years some studies of genetic variation in palm species have been produced, some in the context of habitat fragmentation: Simplicio et al. (2023) suggest that pollination has been maintained by the continuous fruiting and longevity of *Siagrus coronata* in the Brazilian semiarid region during the Late Interglacial Period, exemplifying how reproductive and dispersal traits can maintain gene flow in populations in face of environmental instability. They also found that there is no significant level of inbreeding, though there is moderate genetic structure between populations related to landscape heterogeneity. Fuchs et al. (2023) also found moderate genetic structure in populations of *Chamaedora tepejilote* in mature continuous forest, attributed to limited pollen dispersal by small pollinators (thrips). A congeneric species of our palm, *Astrocaryum aculeatum* has high levels of genetic diversity, but also spatial genetic structure due to short distance seed dispersal (Ramos et al., 2016), a feature that is shared with *A. mexicanum* (though secondary dispersal by rodents may be important in the latter (Brewer, 2001)). In this study, pollen dispersal showed a pattern of isolation by distance. In a second study, Ramos et al. (2022) found genetic structure of *A. aculeatum* that could be associated with the hydrographic basins of two rivers, showing how landscape can influence the genetic structure of this palm, possibly establishing populations at these sites after forest fragmentation (the species settles in deforested areas).

**Figure 1:**
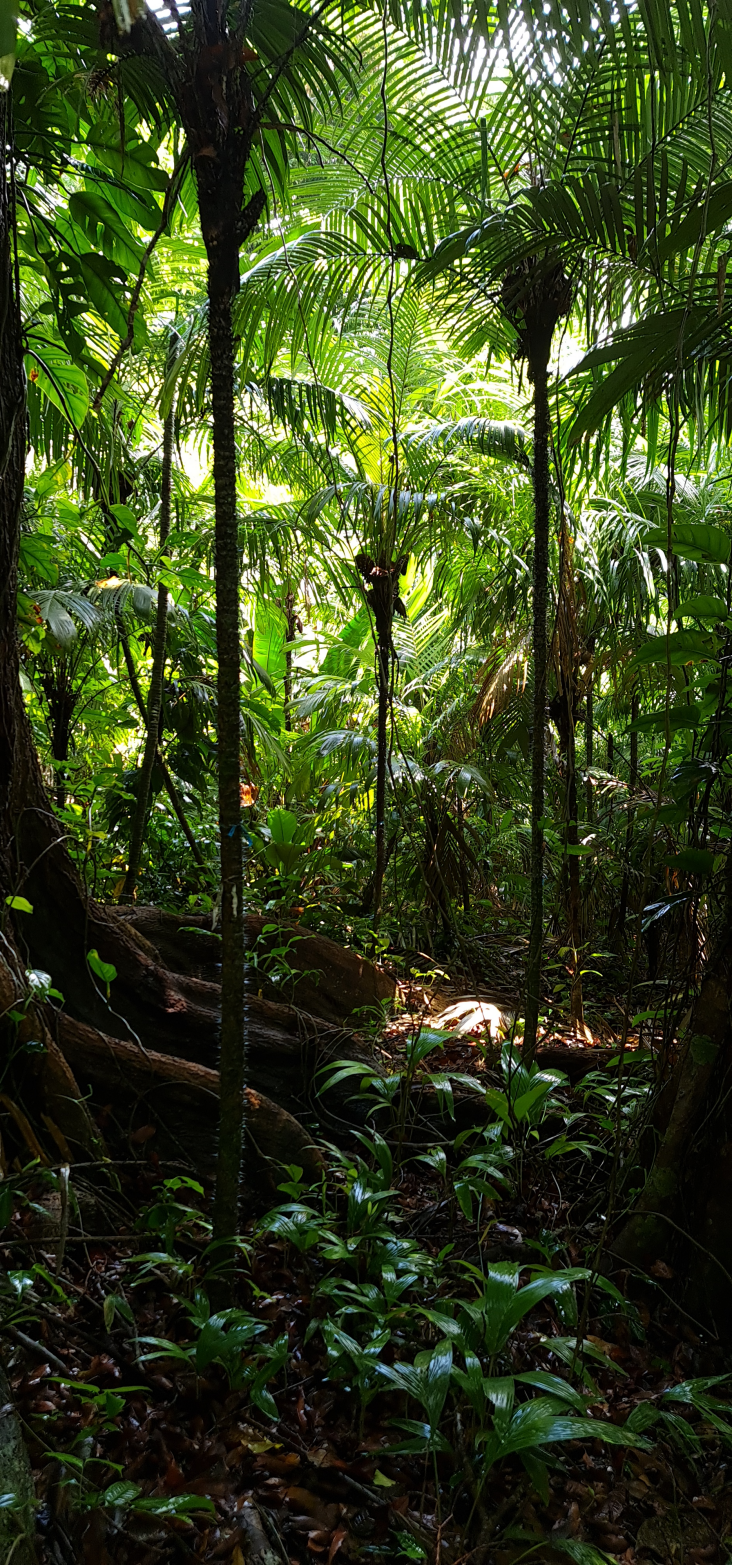
*Astrocaryum mexicanum*. Trees of *Astrocaryum mexicanum* at the Biosphere Reserve Los Tuxtlas. Note the high density of adults and seedlings in the continuous forest (site named Circuito, C1) at low elevation (ca. 100 m a.s.l.). Picture by J. Núñez-Farfán.

The palm tree *Astrocaryum mexicanum* Liebm. (= *Hexopetium mexicanum*; Pintaud et al., 2008) is a long-lived, dominant species in the understory at Los Tuxtlas tropical rainforest, and throughout southern Mexico and northern Central America (Fig. 1) (Piñero, Sarukhán & González, 1977; Vite, 1985). Age at first reproduction is *ca.* forty years old, and most reproductive individuals are two to six meters in height (Sarukhán, 1978), though many reproductive individuals with 1.5 m in height were recorded during the present study; palms can reach up to eight meters in height (J. Núñez-Farfán & J. Juárez-Ramírez, pers. obs., 2015).

The age of *A. mexicanum* individuals can be estimated with precision by their height, because they constantly grow 4.8 cm per year (Vite, 1985; Martínez-Ramos et al., 1989). This species is monoecious and dichogamous–the temporal separation of flowering sexual phases. The flowering peak occurs in April and mature fruits fall to the ground five months later, with a mean dispersal distance range of 0.78–2.35 m from the mother tree (Eguiarte et al., 1993). No fruits are produced by natural self-pollination, though the palm is not strictly self-incompatible when artificial self-pollination is performed (Búrquez, Sarukhán & Pedroza, 1987).

*Astrocaryum mexicanum* is visited by many insect species, mainly small beetles (Búrquez, Sarukhán & Pedroza, 1987; Aguirre & Dirzo, 2008). In the region of Los Tuxtlas, *A. mexicanum* is an ecologically and structurally important plant in the understory of the rainforest, representing 20–61% of total plant density and influencing the diversity of other plants in the understory: a greater dominance of *A. mexicanum* has the effect of reducing the number of individuals of other species in the same area (Piñero, Sarukhán & González, 1977). *Astrocaryum mexicanum* has been subject of detailed, long-term, demographic studies (Piñero & Sarukhán, 1982; Piñero et al., 1986; Martínez-Ramos et al., 1989; Martínez-Ramos et al., 2016), and offers advantages for calibrating time in fragmentation studies compared to other long-lived tropical trees, which usually do not produce annual tree rings (Baker, 2003), allowing us also to determine which palms did establish before and after the onset of fragmentation.

**Figure 2:**
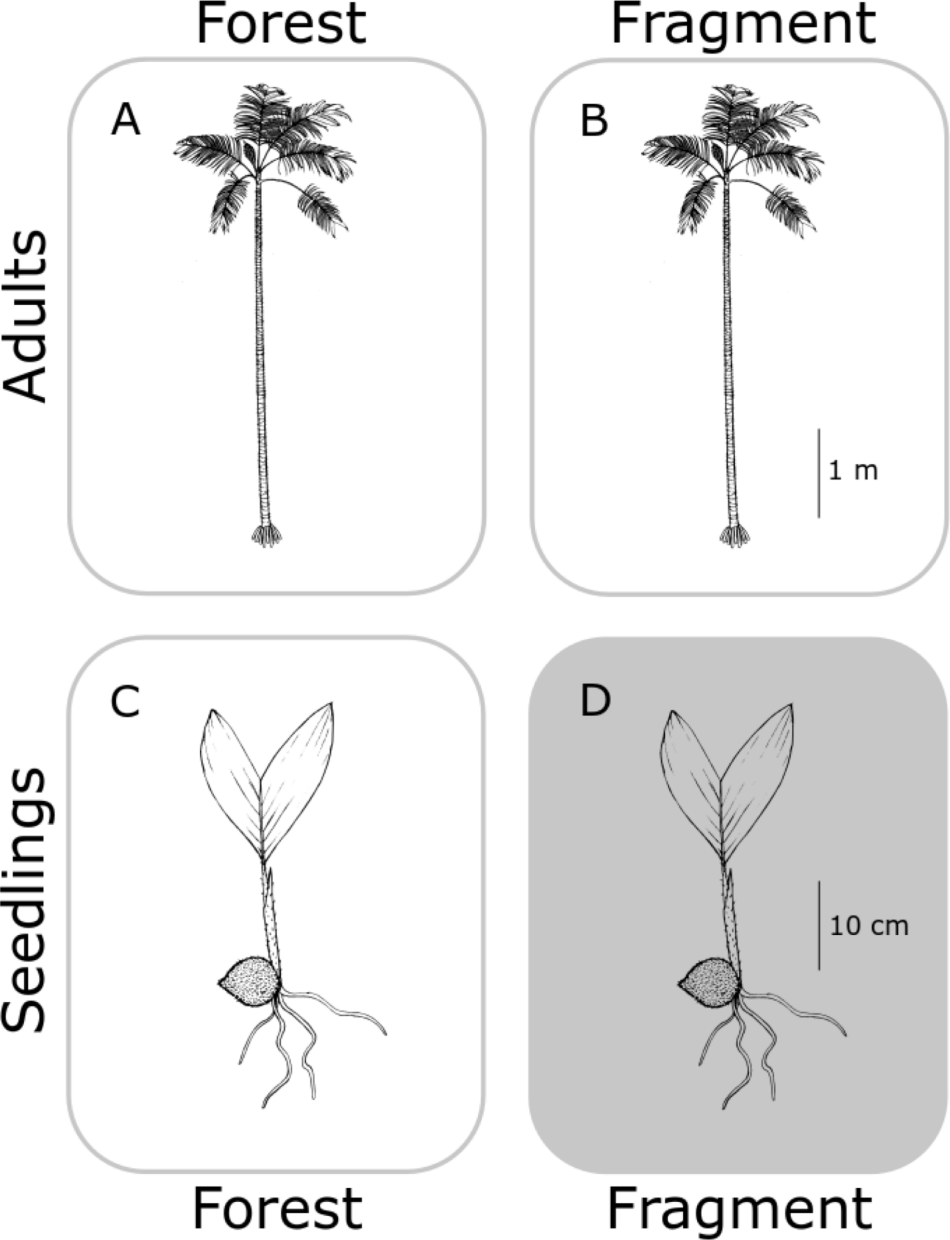
Hypothetical differences in genetic diversity and structure. No genetic differentiation is expected between adult palm trees from continuous (A) and fragmented (B) forests because both predate fragmentation. Seedlings from continuous forests (C) will be similar to adults since they have not been subject to fragmentation effects. Only seedlings in fragments (D) would bear the effects of reduction of population size and isolation, displaying genetic differentiation with respect to the other three groups. Original drawing by L.J. Giraldo Kalil.

In this study, we aimed to determine the genetic structure of both old adults (≥80 years old) and seedlings (1–3 years old) (Fig. 1), for each population, in continuous (C) undisturbed forest and in forest fragments (F), using a set of eight newly developed specific microsatellites (see Supplemental Information 1 and Table S1). We hypothesized that fragmentation restricts gene flow in *A. mexicanum,* increasing genetic differentiation between seedling populations in fragments, and less so (but still significantly) from the adults within site (Fig. 2). In contrast, adults of both type of habitats (undisturbed and fragmented forest) will reflect the historical genetic structure of the region (*i.e.,* little genetic differentiation) since they were born before the fragmentation process. Adults and seedlings from undisturbed forest sites, and adults from fragments, are expected to have comparable genetic variation (or any differences will be due to unaccounted historical factors). Seedlings from fragment sites will be different from any other group (including seedlings from other sites) because they are likely the product of altered mating patterns due to fragmentation. These expectations are depicted in Fig. 2; it must be noted that the “no effect” representation in this figure is only with respect to genetic drift by fragmentation, and not by the original genetic structure of *A. mexicanum*.

Using five isozymic loci, Eguiarte, Pérez-Nasser & Piñero (1992) characterized the genetic structure of populations of *A. mexicanum* of four spatially close populations in the undisturbed forest at Los Tuxtlas (those sampled by Piñero, Sarukhán & González (1977)). Like other tropical trees, *A. mexicanum* has large effective population sizes, high genetic variation and outcrossing rates (Eguiarte et al., 1993), and low genetic differentiation among populations.

Regarding the ecological effects of fragmentation on *A. mexicanum*, Aguirre & Dirzo (2008) suggested that fragmentation diminishes the number of flower visitors, compared to undisturbed habitat. Although *A. mexicanum* is not commercially exploited, it is considered a nuisance due to its spiny trunk and abundance in the understory. For this reason, farmers cut it down in forest fragments to facilitate cattle raising in Los Tuxtlas, adding to its population decline within fragments (J. Juárez-Ramírez & J. Núñez-Farfán, pers. obs., 2015)

The results of this study are relevant to rainforest conservation since *Astrocaryum mexicanum*, a structurally important understory palm, has traits potentially sensitive to forest fragmentation effects like dependence on pollinators, which may not be able to cross the fragmentation matrix, thus affecting population outcrossing rate and reproductive success, and reducing animal fruit (seed) dispersal.

## Methods

### Study site

The region of Los Tuxtlas is located in southern Mexico, in the State of Veracruz, between 18°8′–18°45′N, 94°37′–95°22′W (Eguiarte et al., 1993) and represents the northernmost limit of the tropical rainforest in America (Piñero, Sarukhán & González, 1977). According to the Köppen climate classification, it has a tropical rainforest climate (Af), with a mean annual temperature of 25 °C, annual rainfall between 3,000–4,600 mm, and an altitude range between 0–1,780 m a.s.l. (Gutiérrez-García & Ricker, 2011). Approximately 70% of the original area in the region was covered by lowland rainforest and has been cleared since the second half of the 20th century, at a 4.3%, on average, annual deforestation rate (Dirzo & García, 1992). By 2004 (Guevara, Laborde & Sánches-Ríos, 2004), ca. 1,551.22 km^2^ of rainforest remained, forming a complex fragmented landscape. The structural characteristics of this forest were analyzed by Piñero, Sarukhán & González (1977). It has been suggested that fragmentation in Los Tuxtlas region can promote plant diversity regeneration through negative cascading effects on *A. mexicanum*, as edge effects increase with fragmentation, and this species is not tolerant to intense sunlight (Martínez-Ramos, 1997).

### Sampling and DNA extraction

**Figure 3:**
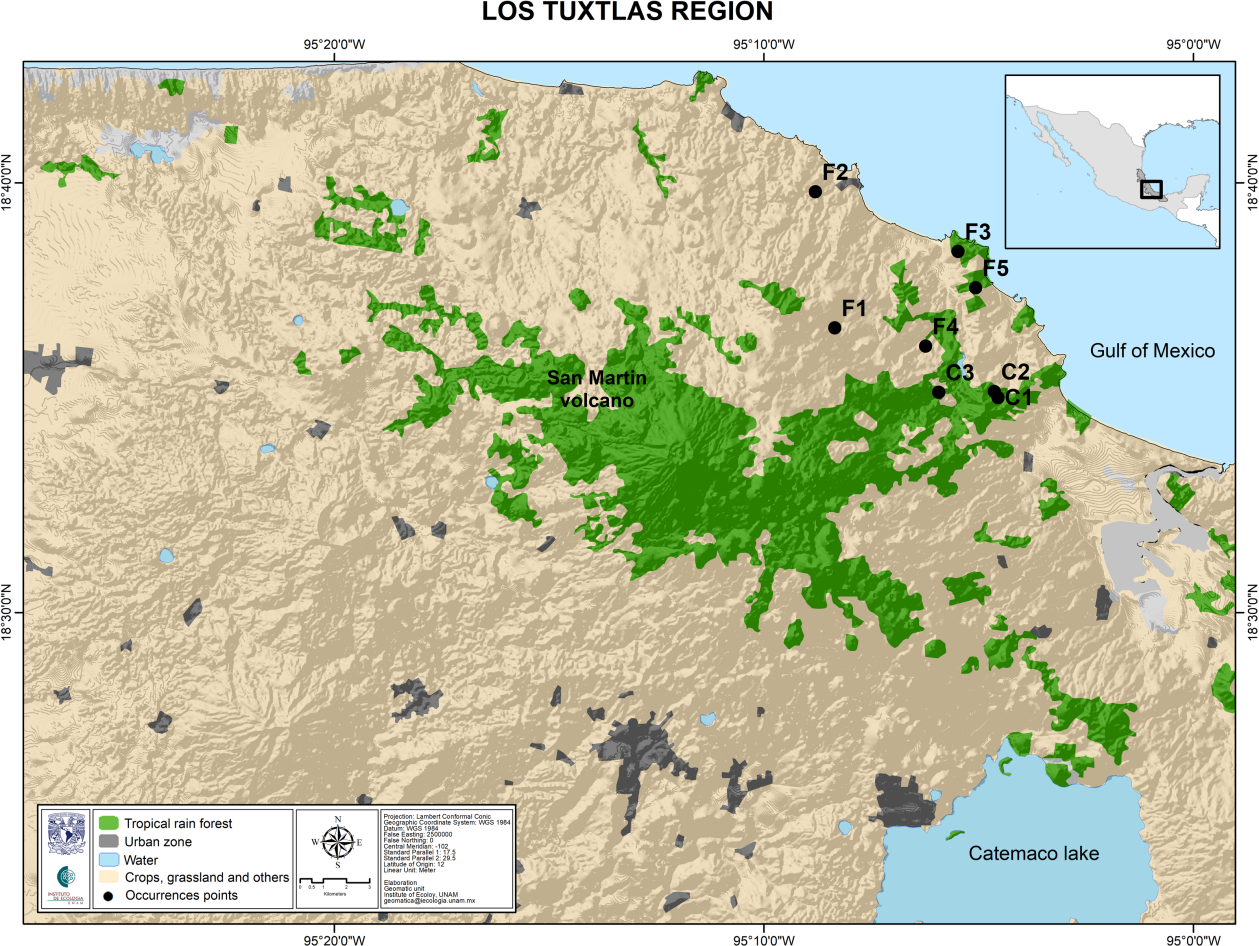
Map of the Los Tuxtlas region depicting the location of the sampled populations. Undisturbed forest populations: C1, Circuito; C2, Lyell; C3, Selva. Fragment populations: F1, Bambú; F2, Cola de Pescado; F3, Borrego Montepío; F4, Ruiz Cortines; F5, Borrego.

The study site was the rainforest of Los Tuxtlas Tropical Research Station, belonging to the National Autonomous University of Mexico. Permission to collect samples of *Astrocaryum mexicanum* for scientific research was granted by the Government of Mexicio (SGPA/DGGFS/712/1596/17). We collected leaf samples from thirty randomly sampled adult palms of at least four meters tall, along a transect (100 m), representing ages of 80–140 years (Fig. 3). These individuals established before the onset of fragmentation (which occurred 60 years ago approximately; see Dirzo & García, 1992). We also collected leaf samples from thirty 2–3 years old seedlings (approximately 5% of leaf area to avoid compromising their survivorship) (Piñero, Sarukhán & González, 1977). Sampled leaves were packaged individually, labeled, and stored in liquid nitrogen for their transportation to the laboratory, and then stored in an ultra-freezer at −80 °C. We extracted DNA from all samples using the protocol for plant DNA extraction developed by Doyle & Doyle (1987). Extracted DNA was stored in a freezer at −20 °C.

### Microsatellite development, amplification and genotyping

We developed eight microsatellite markers as described in the supplementary information. Polymerase chain reaction (PCR) of all samples using the primers developed were made using an Axygen™ Maxygene^®^ thermal cycler, under the following amplification conditions: an initial denaturalization step of 94 °C for 5 min followed by 35 cycles at 94 °C for 1 min, 50 °C for 45 s, and 72 °C for 5 min; finalizing with an elongation step at 72 °C for 5 min. PCR products were mixed in two groups of three and one group of two different microsatellites, considering their molecular weight and the color of the fluorescent dye. The samples were processed in an ABI 3100^®^ (Applied Biosystems, Foster City, California USA) sequencer in the Laboratory of Molecular Biology, at the Institute of Biology UNAM (Mexico City, Mexico). Genotypes were scored visually, using the program Peak Scanner v. 1.0 by Applied Biosystems, and checked for genotyping errors and null alleles with the software Micro-checker v. 2.2.3 (Van Oosterhout et al., 2004).

### Diversity within and between loci, sites and plant generations

Using the program Arlequin v. 3.5.1.2. (Excoffier & Lischer, 2010), we obtained the following genetic parameters for the adults and seedlings of each population:

(a) Number of alleles (*A*): This is the number of alleles that each gene has in the studied populations, which depends on the evolutionary forces that shape genetic variation throughout its history. A gene marker with high number of alleles such as microsatellites has higher temporal and geographical resolution power, so we can infer gene flow and genetic drift changes in the temporal scale of habitat fragmentation. Private alleles (that are only found in one population) were recorded and compared between fragments and undisturbed forest with a Student’s t test.
(b) Observed and expected heterozygosity (*H*_*O*_ and *H*_*E*_), and Hardy-Weinberg equilibrium per locus: the proportion of genotypes of a given locus that are in heterozygous state in a site. The expected proportion of heterozygous genotypes given the frequency of alleles in that locus, assuming an ideal population. A population where both parameters are statistically equal are said to be in Hardy-Weinberg equilibrium. Deviations from this equilibrium (and its direction) can result from violations of an ideal population assumptions. Genetic drift caused by landscape fragmentation can make each subpopulation to differ in these parameters between each other and in relation to the undisturbed habitat. This last one should be near HW equilibrium unless other evolutionary processes are acting.
(c) Linkage disequilibrium between pairs of loci (*D*): assuming independent segregation between loci, pairs of loci of a given genotype should be formed at random. Linkage disequilibrium is the nonrandom association between two pairs of loci in a population. In neutral markers like microsatellites, we can discard selection as the acting force, so observing linkage disequilibrium suggests the action of genetic drift.
(d) Local inbreeding coefficient (*F*_*IS*_). is defined as the probability that two alleles at any given locus are identical by descent in a population. Small populations have a low number of possible mates (and in low dispersing conditions, nearer mates tend to be more related), so this probability increases with each passing generation. This can indicate whether habitat fragmentation reduces mating only to individuals within a fragment.

We estimated *R*_*ST*_, the analogue of *F*_*ST*_ for the stepwise mutation model that microsatellite loci follow (Slatkin, 1995), among pairs of subpopulations. *R*_*ST*_ is defined as the proportion of the genetic variation that resides within subpopulations, with respect to the total. It is a measure of genetic variation. We also performed a locus by locus AMOVA using *R*_*ST*_ as the sum of squared differences with 1,000 permutations. We used GenAlEx 6.5 package for Microsoft Excel to calculate: (a) pairwise relatedness between random individual plants in both cohorts (Lynch & Ritland, 1999) to assess whether fragmentation has led to its increase; (b) Shannon’s mutual information (Sherwin et al., 2006) as another measure of genetic differentiation between populations; and (c) the frequency of private alleles.

Gene flow was separately estimated for seedlings and adults with the method of private alleles (Slatkin, 1985): log_10_[(1)] ≈ *a*log_10_(*Nm*) + *b*, where (1) is the average frequency of private alleles (alleles that are present only in one subpopulation), *a* = 0.505 and *b* =  − 2.44, and *Nm* is the number of migrants per generation to be calculated. According to Slatkin (1985), this method is robust against non-neutrality and low levels of gene flow. Additionally, we obtained Bayesian estimates of migration rate between all pairs of populations using Migrate v3.3.2 (Beerli, 2006; Beerli, 2009).

We used the program STRUCTURE to infer population structure of the whole region (*i.e.,* including all sites) without *a priori* considerations, in order to obtain the number of distinct genetic groups (*K*) as well as the degree of admixture between populations. Ten analyses were performed with *K* = 1 to *K* = 10 for adults and seedlings separately. Δ*K* was calculated and graphed to assess the most likely *K* value for all samples (Evanno, Regnaut & Goudet, 2005).

We estimated ancestral and contemporaneous population size with the program MSVAR v 1.4 (Beaumont, 1999). Although cpSSR markers are preferred for this kind of analysis, previous studies have used nuclear microsatellite data to estimate these parameters successfully in animals (Jorde et al., 1995; Taylor, Sherwin & Wayne, 1994) and plants (Saina et al., 2023). We performed four runs with different prior values for Θ (scaled mutation rate). Due to the lack of information on mutation rate of microsatellites in palms, we used a range of values (10^−3^, 10^−4^, 10^−5^ per generation), which have proven to be adequate in another palm tree (Cibrián-Jaramillo et al., 2009). Since MSVAR is not suited to detect the effects of recent events such as anthropogenic fragmentation (Girod et al., 2011), we did not attempt to run the analysis using the seedlings’ samples or populations in fragments. Initially, each run had 10^5^ steps and was thinned to 20,000 output lines, using different seed and prior values for each run. We ran longer chains if convergence was not reached. We tested the estimates for convergence using the Gelman-Rubin diagnostic (Brooks & Gelman, 1998), as implemented in the CODA package (Plummer et al., 2006) for the statistical software R (R Core Team, 2009).

We simulated a genetic bottleneck using the program BOTTLESIM v2.6 to predict the rate of decay of genetic diversity in a period of 10,000 years after the present. To perform this simulation we used data of adult palms from the site Circuito 1 (C1), in the continuous forest. BOTTLESIM v2.6 (Kuo & Janzen, 2003) incorporates several models of reproductive systems and user-definable level of overlapping between generations. We input realistic parameters, derived from previous studies of *A. mexicanum* on longevity (60 years, which is the mean age of reproductive individuals in the continuous forest) (J. Juárez-Ramírez & J. Núñez-Farfán, unpublished data, 2015), age at first reproduction (30–40 years; Búrquez, Sarukhán & Pedroza, 1987), population size before (Eguiarte et al., 1993) and after (J. Juárez-Ramírez & J. Núñez-Farfán, unpublished data, 2015) population bottleneck (∼1,000 and ∼100–400, respectively), and finally, level of generation overlapping (∼60% seedlings, and ∼40% juveniles and adults) (Piñero, Sarukhán & González, 1977).

## Results

**Figure 4:**
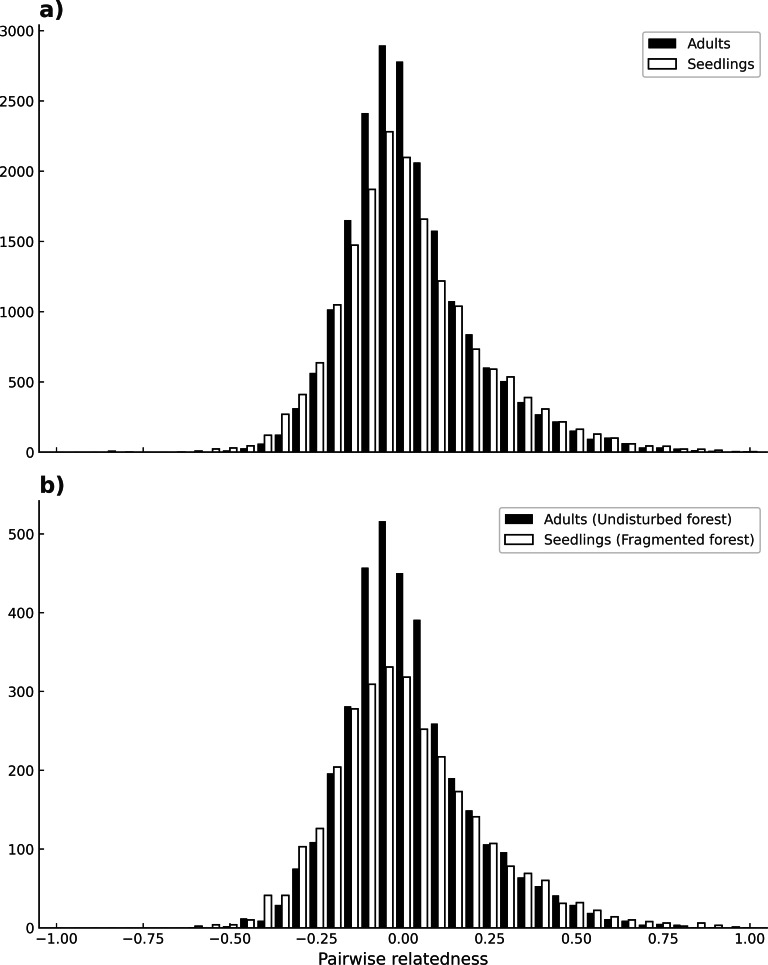
Individual’s pairwise relatedness. Frequency of pairwise relatedness between combinations of all individuals (adults and seedlings), for all loci (A). Pairwise relatedness between all adult individuals in continuous forests and seedlings of fragmented forests (B). The latter comparison is expected to detect differences due to fragmentation.

No evidence of null alleles at any loci was detected by MICRO-CHECKER. Microsatellite loci varied in their degree of polymorphism, ranging from four up to 11 different alleles. The mean number of alleles per locus for seedlings was 5.26 (C. I._95%_ ± 0.409), and for adults is 5.05 (C. I._95%_ ± 0.21) across all loci, and there were no significant differences between adults and seedlings (*W* = 58.5, *p* = 0.3399). In contrast, seedlings had more private alleles (13) than adults (6) (Table 1), and this difference was maintained in both habitats (*t*(7) = 1.19, *p* = 0.03). The mean inbreeding coefficient (*F*) was higher for seedlings (0.13) than for adults (0.08) (*p* ≤ 0.0001) across all sites. For seedlings there was a higher mean inbreeding coefficient value in undisturbed forest (*F* = 0.16), than in fragments (*F* = 0.11) (*χ*^2^ = 0.19, *p* ≤ 0.0001). For adults there was no significant difference between habitats (*χ*^2^ = 0.02, *p* = 1) (Table 1). Loci in significant LD by population and cohort are depicted in Table 1 (see also Table S2). Significant linkage disequilibrium (LD) was observed in most localities (*p* < 0.05), ranging from 0 to 6 pairs of loci with LD. Adults from the sites Bambú and Ruiz Cortínez (fragments) had 6 and 4 pairs of loci with significant LD, respectively. Mean number of loci pairs with LD was higher in adults of fragments (3.4), than in any other site or samples (2–2.3). Mean pairwise relatedness between individuals of all populations was near zero in both adults and seedlings (µ= −0.005 and −0.007, respectively) and the shape of its frequency distribution (of all comparisons between pairs of individuals) was very similar, moderately skewed to the right (SK_P_ = 1.05 for adults and SK_P_ = 0.77 for seedlings; see Fig. 4A). Likewise, the comparison of the frequency distributions of pairwise relatedness of adults from all populations in the continuous forests with that of seedlings from fragments (µ= −0.012 and SK_P_ = 0.87) bore similar results, both in mean value and skewness (Fig. 4B).

**Table 1:** Average estimates of within-population genetic diversity of adult and seedling palms of *Astrocaryum mexicanum*, from continuous, undisturbed, forests and fragments in the biosphere reserve of Los Tuxtlas tropical rainforest. LD, linkage disequilibrium, *F*, inbreeding. Size of the fragments are provided.

Population	Forest size (ha)	Generation	N	# alleles	# Private alleles	Observed *H*	Expected *H*	*F*	Loci in LD (*p* ≤ 0.05)
Undisturbed									
C1. -Circuito	640	Adult	31	5.37	1	0.58	0.61	0.04	2
		Seedlings	26	5.62	1	0.46	0.64	0.28	2
C2.-Lyell	640	Adult	21	5.25	1	0.5	0.6	0.16	3
		Seedlings	27	6	2	0.46	0.58	0.21	5
C3.-Selva	640	Adult	33	5.12	0	0.56	0.59	0.04	1
		Seedlings	28	5.5	3	0.62	0.61	−0.01	0
Mean	640	Adults	28.3	5.25	0.66	0.55	0.6	0.08	2
		Seedlings	27	5.7	2	0.51	0.61	0.16	2.33
Fragments									
F1.-Bambú	17	Adults	26	5.12	2	0.61	0.59	−0.02	6
		Seedlings	23	5.37	2	0.52	0.6	0.13	3
F2.-Cola de Pescado	8	Adults	30	5.12	0	0.47	0.55	0.13	3
		Seedlings	30	5.1	2	0.5	0.58	0.13	2
F3.-Borrego Montepío	9	Adults	20	4.5	1	0.56	0.59	0.04	2
		Seedlings	25	5.25	2	0.52	0.59	0.12	5
F4.-Ruiz Cortines	120	Adults	21	4.65	1	0.49	0.64	0.23	4
		Seedlings	25	4.9	1	0.48	0.56	0.13	1
F5.-Borrego	35	Adults	30	5.25	0	0.58	0.59	0.01	2
		Seedlings	30	4.37	0	0.56	0.59	0.04	0
Mean	37.8	Adults	25.4	4.93	0.8	0.54	0.59	0.08	3.4
		Seedlings	26.6	5	1.4	0.52	0.58	0.11	2.2
Total Mean		Adults	26.5	5.05	0.75	0.54	0.59	0.08	2.87
		Seedlings	26.7	5.26	1.62	0.51	0.59	0.13	2.25
Std. Dev.		Adults		0.3	0.70	0.04	0.02	0.08	1.55
		Seedlings		0.49	0.91	0.05	0.02	0.08	1.98

**Table 2:** Population differentiation. *Rst* values between pairs of populations in continuous and fragmented forests: (a) between adult palms, (b) between seedling palms, and (c) between pairs of adult and seedling populations of *Astrocaryum mexicanum* in both habitats. In (c), the comparison between life stages in a given habitat is indicated with the same letter: A, seedlings from continuous forest *vs.* adults from continuous forest; B, seedlings from continuous forest *vs.* adults from fragments. C, seedlings from fragments *vs.* adults from continuous forest; and D, seedlings from fragments *vs.* adults from fragments. Exact probability is given in parenthesis. Contin, Continuous forest.

(a)										
			ADULTS							
			Continuous			Fragments				
			Circuito	Selva	Lyell	Borrego	Cola de Pescado	Borrego Montepío	Ruiz Cortines	Bambú
ADULTS	Contin	Circuito	—							
		Selva	0.0036	—						
			(0.3693)							
		Lyell	0.1067	0.0981	—					
			(<0.00001)	(<0.00001)						
	Fragment	Borrego	0.004	−0.0025	0.1025	—				
			(0.3964)	(0.5045)	(<0.00001)					
		Cola de Pescado	0.0109	0.0269	0.0396	0.0315	—			
			(0.2252)	(0.081)	(0.1441)	(0.7207)				
		Borrego Montepío	−0.003	−0.0073	0.0936	0.0182	0.0105	—		
			(0.6216)	(0.5675)	(0.018)	(0.1531)	(0.2973)			
		Ruiz Cortines	0.0889	0.0484	0.032	0.049	0.0563	0.0752	—	
			(0.009)	(0.5675)	(0.1621)	(0.081)	(0.09)	(0.09)		
		Bambú	0.0011	0.0082	0.1203	0.0058	0.0228	0.0227	0.0819
			(0.4864)	(0.1891)	(0.018)	(0.2702)	(0.1171)	(0.09)	(0.036)	—

(b)										
			SEEDLINGS							
			Continuous			Fragments				
			Circuito	Selva	Lyell	Borrego	Cola de Pescado	Borrego Montepío	Ruiz Cortines	Bambú
SEEDLINGS	Contin	Circuito	—							
		Selva	0.0055	—						
			(0.2792)							
		Lyell	0.0071	0.0115	—					
			(0.4054)	(0.2522)						
	Fragment	Borrego	0.0259	0.0065	0.0347	—				
			(0.1081)	(0.3153)	(0.072)					
		Cola de Pescado	0.0072	0.0354	0.0563	0.0874	—			
			(0.2364)	(0.3349)	(0.4766)	(0.1354)				
		Borrego Montepío	0.0394	0.0562	0.0204	0.0373	0.0054	—		
			(0.1171)	(0.018)	(0.1261)	(0.063)	(0.3214)			
		Ruiz Cortines	0.0305	0.0235	0.0042	0.0542	0.1223	0.0038	—	
			(0.5231)	(0.2443)	(0.3154)	(0.5496)	(0.0297)	(0.3128)		
		Bambú	0.0154	−0.0045	0.0444	0.0339	0.0215	0.1066	0.0311	—
			(0.2973)	(0.5855)	(0.045)	(0.1081)	(0.090)	(0.009)	(0.1697)	

(c)										
			ADULTS							
			Continuous			Fragments				
			Circuito	Selva	Lyell	Borrego	Cola de Pescado	Borrego Montepío	Ruiz Cortines	Bambú
SEEDLINGS	Continu	Circuito	0.0143^A^	0.0148^A^	0.0774^A^	0.0186^B^	0.0062^B^	0.0006^B^	0.0431^B^	0.0337^B^
			(0.2432)	(0.2252)	(0.036)	(0.1801)	(0.3423)	(0.5765)	(0.1711)	(0.063)
		Selva	0.0102^A^	0.0098^A^	0.0299^A^	0.0111^B^	−0.0031^B^	0.0^B^	0.0329^B^	0.0293^B^
			(0.1982)	(0.1351)	(0.1801)	(0.2072)	(0.5135)	(0.4594)	(0.1261)	(0.063)
		Lyell	0.0188^A^	0.010 ^A^	0.1104^A^	0.0261^B^	0.0307^B^	−0.0165^B^	0.0802^B^	0.0467^B^
			(0.1351)	(0.2522)	(<0.00001)	(0.05)	(0.072)	(0.9459)	(0.018)	(0.009)
	Fragment	Borrego	0.0208^C^	−0.0045^C^	0.0624^C^	−0.0009 ^D^	0.0289^D^	0.0175^D^	0.0114^D^	0.0146^D^
			(0.1621)	(0.6216)	(<0.05)	(0.4774)	(0.1171)	(0.1891)	(0.3333)	(0.1891)
		Cola de Pescado	0.0315^C^	0.0015^C^	0.0723^C^	0.0235^D^	0.0023^D^	0.0015^D^	0.0215^D^	0.0332^D^
			(0.1854)	(0.2644)	(0.3851)	(0. 3526)	(0.2690)	(0.3877)	(0.1647)	(0.2322)
		Borrego Montepío	0.0327^C^	0.0097^C^	0.1801^C^	0.0112^D^	0.0796^D^	0.0253^D^	0.1033^D^	0.0412^D^
			(0.045)	(0.2342)	(<0.00001)	(0.2432)	(0.009)	(0.1531)	(0.027)	(0.036)
		Ruiz Cortines	0.0204^C^	0.0043^C^	0.0842^C^	0.0235^D^	0.0152^D^	0.0034^D^	0.0243^D^	0.0135^D^
			(0.3488)	(0.3217)	(0.2967)	(0.4324)	(0.1755)	(0. 4344)	(0.4111)	(0.1423)
		Bambú	0.0502^C^	0.0499^C^	−0.002^C^	0.0484^D^	0.0017^D^	0.0360^D^	0.0213^D^	0.0694^D^
			(0.081)	(0.063)	(0.5675)	(0.0991)	(0.4594)	(0. 1261)	(0.2612)	(0.018)

Regarding genetic structure, of the 28 possible population pairings, seven pairings of seedling samples, and 11 of adult samples had *R*_*ST*_ values significantly greater than zero (*p* ≤ 0.05). Mean *R*_*ST*_ for seedling and adult pairings were 0.032 and 0.040, respectively, and were not significantly different (*p* = 0.41) (Table 2). Accordingly, Shannon mutual information index had similarly low values for both groups. The AMOVA did not detect differentiation between forest and fragments for both adults and seedlings, and little variation among populations within habitats (continuous and fragments). Most variation was found within populations in both type of habitats (Table 3). There was slightly more variation among populations (not separating by habitat) of adults (4.63%) than of seedlings (3.21%).

**Table 3:** Analysis of molecular variance for adults and seedling of *Astrocaryum mexicanum*, with “population” and “type of habitat” (undisturbed forest and fragments) as factors.

AMOVA for adults			
Variation source	Sum of squares	Variation component	Variation percentage
Between type of habitat	44.86	−0.88	−1.42
Between populations within type of habitat	1,207.7	2.89	4.63
Between populations	1,4465	60.46	96.79

AMOVA for seedlings			
Variation source	Sum of squares	Variation component	Variation percentage
Between type of habitat	101.197	−0.42	−0.67
Between populations within type of habitat	668.513	2.01	3.21
Between populations	1,9014.77	60.94	97.45

**Figure 5:**
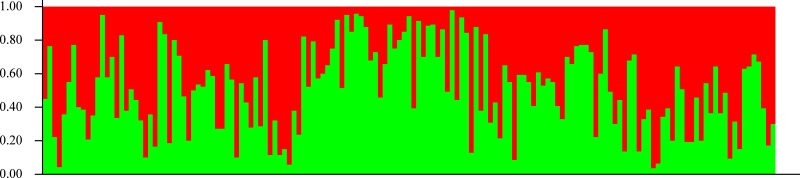
Genetic structure. Lack of population genetic subdivision as depicted by STRUCTURE v2.3.3, for seedlings of *Astrocaryum mexicanum* sampled in eight sites, three in undisturbed forest, and five in isolated fragments. Each vertical line represents an individual and the color composition represents the probability of belonging to a genetic cluster by eight microsatellite loci (see Results).

In agreement with the former results, STRUCTURE analysis showed an optimal *K* value of 2 according to Evanno, Regnaut & Goudet (2005); both seedling and adult populations could not be separated into groups (Fig. 5). According to Pritchard, Wen & Falush (2010), the lowest *K* value that explains most structure must be selected, but a value of 2 did not explain any structure, so it is undistinguishable from *K* = 1.

Gene flow was estimated to be 12.91 migrants per generation for the adult samples, and 15.10 for seedlings with Slatkin’s private alleles method using all populations, whereas Migrate estimated a mean *M* of 24.76 (SD = 16.077, C. I._95%_ = 4.210) for adults and 27.64 (SD = 16.91, C. I._95%_ = 9.89) for seedlings. Number of migrants estimated between pairs of populations ranged from 6.05 to 89.29 with Migrate. Even though *M* obtained by Migrate is larger than with Slatkin’s private alleles, the standard deviation is large enough to consider both estimates equivalent and much larger than Wright’s threshold (1–4) necessary to avoid population divergence.

**Figure 6:**
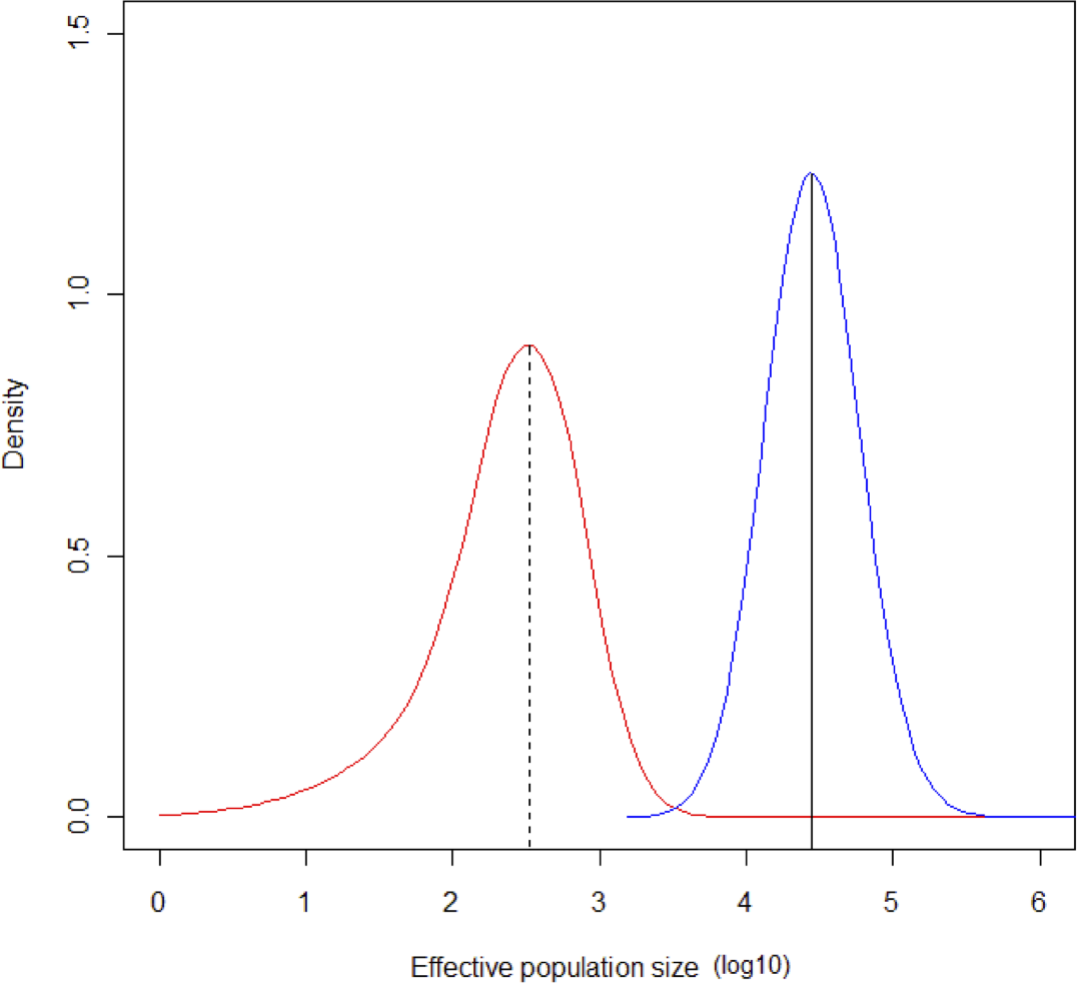
Change in population size during the Last Glacial Maximum. Contemporaneous (dotted line, red probability distribution) and ancestral population size (N_E_) (solid line, blue probability distribution) for Astrocaryum mexicanum at Los Tuxtlas tropical rainforest, as estimated by the program MSVAR v1.4.

The MCMC runs with MSVAR had a high degree of convergence for ancient and current population size, and for mean mutation rate (Point est. = 1.01, upper C. I. = 1.03; Point est. = 1, upper C. I. = 1; Point est. = 1.01, upper C. I. = 1.02, respectively). Estimates of parameter means were as follow: log_10_N_0_ = 4.43 (*σ*^2^ = 0.1508); log_10_N_t_ = 2.5 (*σ*^2^ = 0.4738); log_10_t = 4.4149 (*σ*^2^ = 0.2224). The mode of the posterior distribution for log *r* (*r* = N_0_/N_t_) is 1.768, indicating a clear population decline, occurring at log *t* = 4.414. According to these estimates, a severe bottleneck probably occurred *ca*. 26,000 years ago, in which the population was reduced from an initial 27,000 to 320 individuals (Fig. 6).

**Figure 7:**
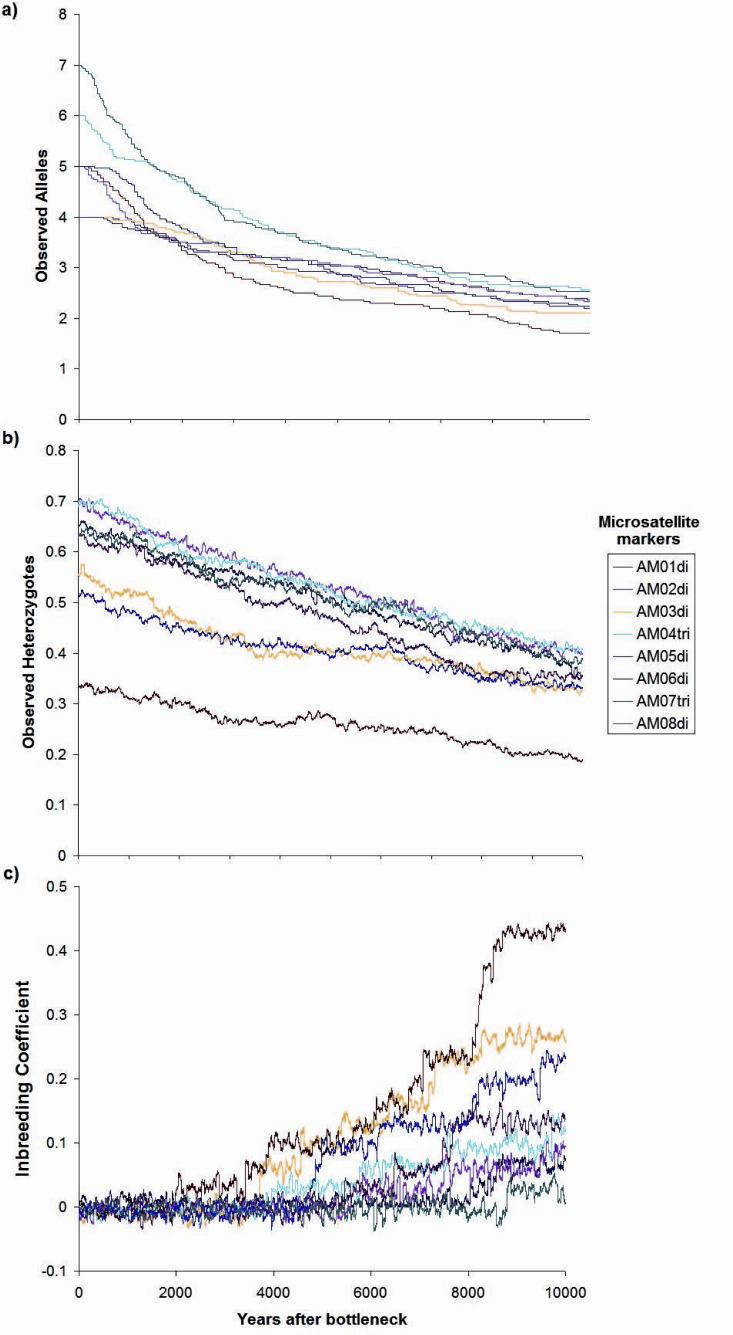
Changes in genetic parameters. (A) Observed alleles, (B) observed heterozygotes, (C) inbreeding coefficient, by genetic drift in a period of ten thousand years since the simulated bottleneck (BOTTLESIM v2.6) for eight microsatellite loci of *Astrocaryum mexicanum* in Los Tuxtlas, Veracruz, with a reduction of N_E_ from 1,000 to 100 individuals.

The simulation of a bottleneck with BOTTLESIM v2.6 using data from one of the continuous populations (Circuito, C1) indicated that we will not be able to detect effects of forest fragmentation (assuming that it truly isolates forest patches) after the first ∼200–500 years, considering the predicted rate of decay of genetic diversity, given the current microsatellite genetic variation (Fig. 7). According to BOTTLESIM, most variation will be lost during the first ∼4,000 years, starting with 4–6 observed alleles, and ending at 1.7–2.5 by 10,000 years (Fig. 7A). Considering an average mutation rate of 10^−4^ per generation (Cibrián-Jaramillo et al., 2009), there would be a new allele per locus every 10,000 years, so we can assume it has little or no role at all in restoring genetic variation. Heterozygosis will be lost in a more linear trend, with the more heterozygous loci presenting a more rapid decline (from 0.7 to 0.4, compared to 0.3 to 0.18 for the less variable locus) (Fig. 7B). The inbreeding coefficient of the three less variable loci will start to increase significantly in ∼3,000–4,500 years after the bottleneck (from an initial ∼0, up to 0.23–0.43 in 10,000 years), and the most variable loci will only have a slight increase or no increase at all (from ∼0 to 0–0.11) (Fig. 7C). If gene flow is maintained between remnant populations, diversity loss will be buffered.

## Discussion

*Astrocaryum mexicanum*’s populations from southeast Mexico show high population connectivity: a high number of migrants per generation have led to low differentiation between sites, a fact that clearly indicates the effectiveness of *A. mexicanum* pollinators to move through long distances, in accordance to results obtained by Eguiarte, Pérez-Nasser & Piñero (1992). Trees born before and after fragmentation bore no significant differences, either with estimations derived from Slatkin’s private alleles or with MIGRATE’s bayesian methods, but differences in the number of migrants between sites are very large, with values ranging from 6.05 to 89.29 migrants. This is congruent with the low but significant population differentiation detected among sites, and *R*_*ST*_ values are similar to those found by Eguiarte, Pérez-Nasser & Piñero (1992) in the continuous forest. Accordingly, estimations of pairwise relatedness have a mean value near zero on both adults and seedlings, and the frequency distribution of these pairwise comparisons between individuals have a nearly identical shape and slight skewness to the right (towards negative relatedness values) (Fig. 3A). Likewise, comparisons between fragment seedlings and undisturbed forest adults, which according to our prediction would be highly differentiated (cf. Fig. 1) show remarkably similar frequency distribution of pairwise relatedness (Fig. 3B), suggesting that pollinators can effectively cross of the fragmentation matrix. This has implications for conservation and genetic restoration of degraded forests, as even fragmented populations can be sampled for seeds and pollen without danger of having a bias for related individuals. The results agree with the findings in other insect-pollinated palm species in the same rainforest (Luna, Epperson & Oyama, 2005), as well as other tropical trees of the region (Pérez-Nasser, Eguiarte & Piñero, 1993; Cibrián-Jaramillo, 2017).

Most genetic diversity is found within subpopulations and of the same magnitude as in other tropical palms for microsatellite loci, like *Euterpe edulis* (*H*_*e*_ and *H*_*o*_ of 0.76 and 0.70, respectively; Gaiotto, Grattapaglia & Vencovsky, 2003) and *Chamaedora ernesti-augusti* (*H*_*e*_ of 0.67 and *H*_*o*_ of 0.39; Cibrián-Jaramillo et al., 2009).

A larger number of private alleles was found in seedlings than in adults, regardless of habitat, a result similar to a pattern found in another study in the tropical tree *Koompassia malaccensis*. As microsatellites are adaptively neutral, and assuming no linkage to genes under selection, only gene flow, genetic drift and mutation can account for this pattern. It is possible that mutation can increase private alleles in progeny, and later genetic drift may erase some of these alleles as the palms grow and some of them die from natural and unnatural causes. Low frequency alleles (such as private alleles in this case) have a higher probability of disappearing.

However, we should note that these estimates of gene flow represent a combination of historical and contemporary events, and it is uncertain whether contemporary gene flow has been diminished by fragmentation. The lack of differentiation among sites does not support the hypothesis that gene flow has been diminished, but it is possible that the number of generations since the onset of fragmentation in the region has not been large enough as to detect its effects on genetic structure of *A. mexicanum,* since reproductive individuals born after fragmentation might constitute a small fraction of adults. If it is determined that high connectivity between fragments exists, this would underscore the importance of maintaining patches of vegetation, even if small, to certain species of plants and their pollinator ensemble.

As other palm species, *A. mexicanum* bears significant inbreeding. Since *A. mexicanum* does not produce fruits by self-pollination (Búrquez, Sarukhán & Pedroza, 1987), it is likely that most inbreeding is due to mating between relatives (biparental inbreeding). This can be tested by estimating the difference between the multilocus and single locus outcrossing rates. Higher inbreeding in seedlings than in adults would support the hypothesis that in these populations of *A. mexicanum* there is selection against homozygotes (Eguiarte, Pérez-Nasser & Piñero, 1992). An interesting result is that seedlings are more homozygous in the continuous forest than in fragments. This suggests that harsher environmental conditions (in terms of humidity, light incidence, temperature and wind speed) in fragments of Los Tuxtlas (Suárez-Montes, Chávez-Pesqueira & Núñez Farfán, 2016) result in stronger selection against homozygotes at earlier life stages, surpassing the effect of a hypothesized increased inbreeding. A similar result has been reported in the herb *Arnica montana* in fragmented forests of Germany: populations of this plant species even have an excess of heterozygotes, and the authors suggested that heterozygotes are more resistant to the harsher post-fragmentation conditions (Kahmen & Poschlod, 2000). Although we used neutral markers in this study, their homozygosity is linked to biparental inbreeding which affects allele distribution in all genes, and self-incompatible plants like *A. mexicanum* tend to present higher inbreeding depression due to genetic load. Thus, we assume that homozygous individuals for the neutral markers are also more homozygous for non-neutral markers and therefore are more susceptible to selection against homozygotes. To test this hypothesis in *A. mexicanum*, multilocus genotypes must be assessed for germination and seedling survivorship in fragments and undisturbed forests, a type of study called heterozygosity-fitness correlation. Szulkin, Bierne & David (2010) make a review of the value for such approach, though it is not without limitations (Kirk & Freeland, 2011).

It remains to be determined whether reduced outcrossing takes place in fragments, although genetic data from seedlings does not seem to support this notion. Although selection may maintain heterozygosity in some species, stronger selection against homozygotes may reduce the number of future mates and compromise population viability, if accompanied by low pollinator visit rates and barriers to long distance pollination.

There are many loci pairs in linkage disequilibrium for adults in fragments, suggesting that fragmentation may have caused a biased sampling of allele combinations in *A. mexicanum*’s genetic diversity. As these adults reproduce, linkage disequilibrium would break down, resulting in similar values of this parameter in seedlings of disturbed and undisturbed habitats. This explanation stems from the study of *Betula maximowicziana,* where adults of sites subject to a bottleneck by a fire had higher LD than adults in undisturbed sites, whereas seedlings showed no significant differences between sites (Uchiyama et al., 2006).

Recent meta-analyses have found that forest fragmentation has a negative effect in woody long lived plants, with similar traits than those of *A. mexicanum*. González, Gómez-Silva & Ramírez (2019) found that the negative effects in allelic richness but not for *H*_E_ are related with self-incompatibility, commonness, and being tropical. Schlaepfer et al. (2018) also showed that forest habitat has negative effects of fragmentation on genetic diversity for woody plants, but not for herbs. Vranckx et al. (2011) confirms the negative effect of fragmentation on woody plants, especially for those with insect pollinated like *A. mexicanum*, which is pollinated by small beetles. Furthermore, recent studies on various palm species have found moderate genetic structure, both in continuous forest and areas isolated by landscape patterns (Ramos et al., 2016; Ramos et al., 2022; Fuchs et al., 2023; Simplicio et al., 2023). A possible reason of why a plant such as *A. mexicanum* with seemingly so many traits associated to susceptibility to fragmentation does not show negative effects in allelic richness, is offered in the work of Schlaepfer et al. (2018): time since fragmentation seems to be an important factor. In general, they found negative effects of fragmentation (number of alleles and heterozygosity) in forest fragments older than 50 years. Thus, although we did not detect negative genetic effects of fragmentation in *A. mexicanum* it seems that their likelihood would increase with the age of fragments

Even though current genetic structure of *A. mexicanum* cannot be explained by forest fragmentation, the Bayesian analysis performed with MSVAR suggests that its current genetic diversity was influenced by a severe bottleneck ∼26,000 years ago. This corresponds to the beginning of the Last Glacial Maximum (LGM), which is the period between 26,500–19,000 years ago (Clark et al., 2009), and CA 16,900 years ago the abrupt deglaciation began, stabilizing approximately 10,000 years ago to preindustrial temperatures (Osman et al., 2021). The LGM represents the peak of the last glacial period, when the ice sheets had their maximum coverage extension, provoking the diminishing and retreat of the tropical rainforest vegetation to Central America (Ray & Adams, 2001). In Mexico, the last glacial period induced a mean temperature drop of 4 °C in the Gulf Coastal Plain, reducing the extension of the tropical climate (Sarukhán, 1977). In Los Tuxtlas region, the tropical climate was limited to two refugia, north and south of the current biosphere reserve location (the lower Bank of the Papaloapan River, and the Minatitlan-Coatzacoalcos zone, respectively) (Emiliani, 1956). Such contraction and expansion of a population may have generated low contemporary genetic structure, since colonizers would have come from these refugia, homogenizing genetic diversity. Further contemporary contraction-expansion events associated with volcanic activity in the region, approximately 150 years ago (Martín del Pozzo, 1997), might have also played a role in this phenomenon as well. Other species in the region may present the same contraction-expansion pattern than *Astrocaryum mexicanum* (see Suárez-Montes, Chávez-Pesqueira & Núñez Farfán, 2016) and may have a strong effect in present genetic structure that supersedes any effect from contemporary fragmentation.

Although we did not detect an effect of forest fragmentation on within-site genetic variation of *A. mexicanum*, a bottleneck simulation of the same magnitude as in some forest fragments, showed that genetic impoverishment can be detected this century, though some parameters will take much longer to change. This situation is expected under the relatively optimistic, but sadly unsupported assumption that current forest cover will not decrease any further; the real scenario is likely to be more detrimental. Having a species’ demographic parameters, life history and contemporary genetic variation is important to be able to make accurate simulation-based predictions. Only if current rates of gene flow between patches can be maintained, genetic diversity and low population structure could be maintained indefinitely, giving opportunity for realistic approaches to conservation, for instance, by promoting preservation of natural forest corridors (see Figueroa-Esquivel et al., 2010).

## Conclusions

There is no discernible effect of habitat fragmentation in the genetic structure of the palm tree *Astrocaryum mexicanum* population in Los Tuxtlas region in Mexico. Microsatellite markers show a history of high connectivity in this area and overall high genetic diversity. Simulations with precise data of life history and genetic diversity predict decay of genetic diversity parameters, some of them detectable this century (observed alleles), but some taking thousands of years (increase of inbreeding coefficient). Bayesian analysis suggests current genetic diversity and structure is strongly influenced by contraction and recolonization during the Last Glacial Maximum ca. 10,000 YBP. There is some evidence suggesting harsher environmental conditions in fragments selecting against homozygotes. Further work is needed to assess whether fragmentation is promoting increased selfing, as it represents a more contemporary effect that may not be captured by assessing genetic parameters such as *F* or *H*_O_.

## Supplemental Information

10.7717/peerj.19784/supp-1 Supplemental Information 1 Development of microsatellite loci for *Astrocaryum mexicanum* Microsatellite isolation and polymorphism. Primers of microsatellite loci of Astrocaryum mexicanum. Pairs of microsatellite loci in linkage disequilibrium.

## Additional Information and Declarations

### Field Study Permissions

The following information was supplied relating to field study approvals (*i.e.*, approving body and any reference numbers): Secretaria de Medio ambiente y Recursos Naturales, Gobierno de México. The sampling site is a station of the University of Mexico.

### Data Availability

The following information was supplied regarding data availability: Data is available at: Juarez-Ramirez, Jorge Octavio (2025). astrocaryum1.arp. figshare. Dataset. https://doi.org/10.6084/m9.figshare.20277879.v1.
