# Supplementary material for: Past and present genetic structure of the tropical rainforest palm Astrocaryum mexicanum: effects of anthropogenic fragmentation
Source: PeerJ. 2026 Jan 13;14:e19784. doi: 10.7717/peerj.19784 (PMC12810366; doi:10.7717/peerj.19784)
Supplement: Supplemental Information 1 — Microsatellite isolation and polymorphism. Primers of microsatellite loci of Astrocaryum mexicanum. Pairs of microsatellite loci in linkage disequilibrium. [file peerj-14-19784-s001.docx]

Supplementary Material from:

**Past and present genetic structure of the tropical rainforest palm *Astrocaryum mexicanum*: Effects of anthropogenic fragmentation**

Jorge Octavio Juárez-Ramírez^1^, Juan Núñez-Farfán^2^

*Laboratorio de Genética Ecológica y Evolución, Departamento de Ecología Evolutiva, Instituto de Ecología, Universidad Nacional Autónoma de México; Apartado Postal 70-275, México, Distrito Federal 04510, México.E-mail adresses:*^1^jjuarezr@gmail.com; ^2^farfan@unam.mx (corresponding author)

**Index**

**Page**

Development of microsatellite loci for *Astrocaryum mexicanum*. 2

Microsatellite isolation 2

Microsatellite polymorphism 2

Conclusions 4

References 4

Acknowledgements 5

**Table S1.** Primers of microsatellite loci of *Astrocaryum mexicanum* 6

**Table S2.** Pairs of microsatellite loci in linkage disequilibrium 7

**Microsatellite isolation**

Total DNA was extracted from one individual of *Astrocaryum mexicanum* using the DNeasy plant mini kit protocol (Qiagen, Valencia, CA), following the manufacturer´s protocols. To increase the probability of detecting microsatellites, we followed the enrichment procedure of [Glenn & Schable (2005)](http://www.sciencedirect.com/science/article/pii/S003140561000106X#bib0065), with some modifications. Briefly, the DNA was digested with the restriction enzyme RsaI (New England Biolabs) and simultaneously ligated to double-stranded Super SNX linkers, denatured and hybridized to biotinylated microsatellite oligonucleotide mixes [mix 2=(AG)12, (TG)12, (AAC)6, (AAG)8, (AAT)12, (ACT)12, (ATC)8; mix3= (AAAC)6, (AAAG)6, (AATC)6, (AATG)6, (ACAG)6, (ACCT)6, (ACTC)6, (ACTG)6; mix 4= (AAAT)8, (AACT)8, (AAGT)8, (ACAT)8, (AGAT)8], then, captured on magnetic streptavidin beads (Dynal). Unhybridized DNA was washed away, and remaining DNA was eluted from the beads, amplified in polymerase chain reactions (PCR) using the forward SuperSNX24 as a primer, and cloned with TOPO-TA Cloning Kits (Invitrogen). Inserts from a total of 272 clones were PCR amplified and the enriched libraries were sequenced on a next-generation sequencer at the Georgia Genomics Facility in Athens, GA (454 Life Sciences, Roche, Branford CT) using titanium chemistry following standard Roche 454 library protocols. The enriched *A. mexicanum* library was pooled with other enriched libraries that had unique Simple X linkers, such that each SimpleX barcode was used only once in any given pool. Once pools are formulated, each pool was processed using standard Roche 454 library protocols, where the 454 sequencing primers and MID-tag barcodes were ligated on to each fragment. Each SimpleX pool received a different MID-tag barcode for subsequent identification. Libraries were then quantified and sequenced following standard Roche 454 protocols. Microsatellites were identified using MsatCommander version 0.8.1 (Faircloth, 2008) and primers designed with Primer3. One primer from each pair was modified on the 5´end with an engineered sequence (CAG tag 5´CAGTCGGGCGTCATCA-3´) to enable use of a third primer in the PCR (identical to the CAG tag) that was fluorescently labelled for detection.

**Microsatellite polymorphism**

Primer pairs were tested for amplification and polymorphism using DNA obtained from individuals sampled in a population in the undisturbed continuous forest at the Los Tuxtlas Reserve, and the amplified PCR products were then separated on 2% agarose gels (Invitrogene^TM^). After excluding loci that did not amplify, we selected eight potential polymorphic loci and marked these with fluorescent labels (Table S1). The PCR amplification was carried out in a 20-μL reaction containing 2 μL of 10× PCR buffer (KCl 500 mM, Tris-HCL pH 8.3, gelatin 100 μg/mL, 1% triton, bovine serum albumin [BSA] 1.5 mg/mL), 1 μL of MgCl2 (30 mM), 2 μL of dNTPs (0.2 mM), 2 μL of DNA, 0.5 μL of each of the two primers (10 mM), 0.5 μL of *Taq* DNA polymerase (5 U/μL), and 12 μL of water (BIOTECMOL, Mexico City, Mexico), performed on a Maxygen- thermal cycler (Axygen-Corning Life Sciences, USA), using the following conditions: 94°C for 5 min; followed by 35 cycles of 94°C for 1 min, at temperatures between 50°C for 45 sec, and 72°C for 1 min; and a final extension step of 72°C for 10 min.

To encompass the most genetic diversity of *A. mexicanum* in the Los Tuxtlas rainforest, we collected leaf tissue of 32 individuals from Los Tuxtlas Reserve population. Genomic DNA was extracted following the cetyltrimethylammonium bromide (CTAB) MiniPrep protocol (Doyle & Doyle, 1987). We selected the polymorphic loci with labelled primers (6-FAM, VIC, NED, Applied Biosystems, Foster City, California, USA) (Table S1). Each microsatellite amplification PCR mixture (20 μL) contained 2 μL of DNA template (20 ng), 0.2 μL of each fluorescent-labeled forward primer (0.2 μM), 0.2 μL of each reverse primer (0.2 μM) were done with similar conditions as above. To check amplification, 5 μL of the PCR products were subjected to electrophoresis in a 2.0 % agarose gel with 1× TBE buffer and stained with ethidium bromide. For genotyping, 5 μL of each PCR amplified product were diluted in 10 μL of water. One or two microliters of these PCR products (20–50 ng) were run on ABI Prism 310 and ABI 3730xl (Applied Biosystems) automated capillary sequencers; allele sizes were scored manually using Gene- Scan 500 LIZ Size Standard (Applied Biosystems) in GeneMarker version 2.4.0 (SoftGenetics LLC, State College, Pennsylvania, USA).

Of the 20 primers tested, 8 were polymorphic. For each polymorphic locus, we calculated the number of alleles (*A*), observed heterozygosity (*H*o), and expected heterozygosity (*H*e); tests of deviations from Hardy–Weinberg equilibrium (HWE) and linkage disequilibrium (LD) were performed using the software Arlequin version 3.5.1.3 (Excoffier & Lischer, 2010). Results of these tests are to be found in the main article (*cf*. Table 1).

**Conclusions**

The development of these microsatellite markers will be a great aid in population genetics studies of *Astrocaryum mexicanum*, as well as other closely related. This is relevant because palms have been scarcely studied (understory palms). The eight polymorphic microsatellite markers we developed will aid in the study of *A. mexicanum* genetic structure and mating system. Microsatellite reported here, will help to study the genetic structure in fragmented populations of the rainforest, and tackle not only basic questions but applied ones in evolutionary biology.

**References**

Doyle J, Doyle J. 1987. A rapid DNA isolation procedure from small quantities of fresh leaf tissues. *Phytochemical Bulletin* **19**: 11–15

Excoffier, L, Lischer HEL. 2010. Arlequin suite ver. 3.5: A new series of programs to perform population genetics analyses under Linux and Windows. *Molecular Ecology Resources* **10**: 564–567

Faircloth BC. 2008. MSATCOMMANDER: detection of microsatellite repeats arrays and automated, locus-specific primer design. *Molecular Ecoogy Notes* **8**: 92–94

Glenn TC, Schable NA. 2005. Isolating microsatellite DNA loci. *Methods Enzymology* **395**:202–222

Holland MM, Parson W. 2011. GeneMarker® HID: A reliable software tool for the analysis of forensic STR data. *Journal of Forensic Sciences* **56**: 29–35

**Acknowledgments**

The authors thank the Los Tuxtlas Biological Research Station for logistics support; S. Lance and the University of Georgia Savannah River Ecology Laboratory for sequencing and developing primers; and to Laura Márquez-Valdemar, for assistance in obtaining genetic data. This paper constitutes an integral part of the Doctoral Thesis of Jorge O. Juárez-Ramírez for the Graduate Program in Biological Sciences, at the Universidad Nacional Autónoma de México (UNAM).

**Table S1.** Primers of microsatellite loci of *Astrocaryum mexicanum*. GenBank accession number of microsatellite amplification products.

| Locus  name | Sequence of forward and  reverse primers | Locus  size (bp) | GenBank  accession number |
| --- | --- | --- | --- |
| di1002 | TAAGCAGGGAAGATGAAATC TTGTCTCCCTGGTTGAATC | 357 | KX822148.1 |
| di1154 | GATTGTGCGAGATTGAACTC CGTCTCCAATAACATCCAC | 215 | KX790328.1 |
| di1462 | CCCATCCGTATTATGAAGTC TTTGACGTCTTGTGCTCAG | 179 | KX790325.1 |
| di1802 | CCTCATCATTTAAACCTTGAC CAAATATTCCACCCATCATG | 123 | KX822146.1 |
| di1966 | GTCCAGATCCAGCCTTAAC CATGATCAAACTGACTGGTTAC | 228 | KX790327.1 |
| tri966 | TTATGCTTTGAGGCCTTATC CTTAACATTGGACCAAGGAG | 172 | KX822147.1 |
| triDJWN3 | GGTGTTGTGTTGCTGACTTC GCAGCAATGAATGAGGTAG | 161 | KX822151.1 |
| tri1681 | TTGGAGTTCATTGAGGAAAC CTAAGGCTCAGTTTCCAAAG | 339 | KX790326.1 |

**Table S2.** Pairs of microsatellite loci in significant linkage disequilibrium (*p* < 0.05) by population (see Table 2 in the main text).

|  | Undisturbed forest | |  |  |  |
| --- | --- | --- | --- | --- | --- |
|  | Circuito 1 | Lyell | Selva 2 |  |  |
| Cohort |  |  |  |  |  |
| Adults | di1154 & di1681 | di1462 & di1802 | di1802 & tri966 |  |  |
|  | tri966 & di1002 | di1802 & di1002 |  |  |  |
|  |  | tri966 & di1002 |  |  |  |
|  |  |  |  |  |  |
| Seedlings | triDJWN & tri966 | di1462 & di1802 | none |  |  |
|  | di1802 & di1966 | di1462 & di1681 |  |  |  |
|  |  | triDJWN & di1002 |  |  |  |
|  |  | di1802 & di1002 |  |  |  |
|  |  | di1802 & di1681 |  |  |  |
|  |  |  |  |  |  |
|  | Fragments |  |  |  |  |
|  | Cola Pescado | Borrego | Borrego M | Ruiz C | Bambú |
|  |  |  |  |  |  |
| Adults | di1154 & di1681 | triDJWN & di1681 | di1154 & triDJWN | di1154 & di1802 | di1462 & di1681 |
|  | di1154 & di1802 | di1966 & di1681 | triDJWN & di1966 | triDJWN & di1802 | triDJWN & tri966 |
|  | di1802 & di1966 |  |  | di1462 & tri966 | triDJWN & di1002 |
|  |  |  |  | di1462 & di1681 | tri966 & di1966 |
|  |  |  |  |  | tri966 & di1002 |
|  |  |  |  |  | di1966 & di1681 |
|  |  |  |  |  |  |
| Seedlings | di1154 & di1802 | none | di1154 & triDJWN | triDJWN & di1802 | triDJWN & tri966 |
|  | di1802 & di1002 |  | di1462 & di1966 |  | di1802 & di1966 |
|  |  |  | di1802 & di1966 |  |  |
|  |  |  | di1154 & di1002 |  |  |
|  |  |  | di1154 & di1681 |  |  |
